# Social networks as pathways of political mobility in Portuguese governments

**DOI:** 10.1371/journal.pone.0355224

**Published:** 2026-08-19

**Authors:** Rafael Borges, Carolina Shaul, Flávio L. Pinheiro, Bruno Damásio

**Affiliations:** NOVA Information Management School (NOVA IMS), Universidade NOVA de Lisboa, Lisboa, Portugal; Thammasat University, THAILAND

## Abstract

Political mobility in executive appointments is often explained through party affiliation and career trajectories, yet relational embeddedness across institutional arenas may also characterise those who reach ministerial office. We examine, retrospectively and in an exploratory manner, whether the social networks formed by co-participation in Portuguese governmental and parliamentary venues are associated with ministerial appointments. Using web-scraped data on the composition of the Constitutional Governments and the Portuguese Parliament between 2015 and 2024, complemented with the Registers of Interests of government and parliament officials, we construct an individual-venue bipartite network and its projection onto individuals. We estimate the cumulative network of Governments XXI-XXII and, within each policy-domain, retrospectively screen potential candidates for the XXIII Government by ranking actors in the top five positions of at least one centrality measure (betweenness, reach, closeness, weighted closeness, or eigenvector), computed on portfolio-specific subgraphs and restricted to nodes reachable from the Prime Minister. With the identification of 9 out of 16 in-network ministerial appointments, this procedure outperforms both a random selection baseline (mean recall 0.6%) and a career-ladder heuristic based on prior secretarial experience in the same domain (recall 12.5%), consistent with network centrality being informative about the appointment process in stable, well-populated policy-domains. We assess whether Ministers exhibit systematically higher institutional reach than non-ministerial actors using bipartite degree centrality, visualised via a log-scaled KDE, and validated with non-parametric tests (10,000-iteration permutation tests and Mann-Whitney U tests). We decompose connectivity by venue type (e.g., Government, Parliament, Political Party) using a categorical profile comparison and cosine similarity. Ministers display higher bipartite degree centrality than non-ministerial actors (mean 0.0200 vs. 0.0105; difference 0.0096; permutation p-value = 0.021; Mann-Whitney p-value < 0.001). Categorical profiles diverge substantially (cosine similarity 0.494), with significant gaps concentrated in Government and Parliament venues, while Parliamentary Bodies show no meaningful difference (p-value = 0.919). Venue-specific projected network analysis further reveals that the minister-non-minister centrality gap is largest within the political party network, where ministers score nearly double on eigenvector centrality (+96.0%; permutation p-value = 0.003), pointing to elite positioning within partisan structures.

## Introduction

Political mobility refers to the movement of an individual or a group between positions within the political system, possibly mirroring shifts in influence and voter behaviour. The study of political mobility ideally can reveal power dynamics, political preferences, allegiances, and representativeness over time.

Network analysis offers a systematic framework for examining relational structures, allowing the mapping of interactions within and across governmental institutions. For example, [1] applied network analysis to study committee membership in the United States House of Representatives, identifying a hierarchical structure between committees and correlation between these and Representatives’ political positions. [2] used 200 years of historical data on the Greek government to investigate possible relationships among ministerial cabinets, based on the number of Ministers who served in them. Another approach to network analysis for studying mobility considers the “occupational network”, where units of analysis are occupations or roles, from which, for example, [3] estimated occupational mobility (i.e., changes across occupations) in the U.S. labour market. [4] explores this approach for the case of the U.S. government’s composition by studying personnel transitions across governmental executive and corporate functions, treating these occupations as nodes. In [5], political mobility in Portugal is also studied from the perspectives of occupational networks and policy-domain networks (i.e., networks in which nodes refer to different ministerial cabinets).

On the other hand, network analysis of governmental structures can also be pursued through social networks, in which individuals are linked based on whether they directly share a relationship. For instance, using this metric, [6] explored the relationship between Ministers regarding collaboration and co-sponsorship of legislative proposals in Brazil. In the Portuguese case, [7] employed social network analysis to investigate relationships between Ministers and Secretaries of State and the private sector, considering connections such as shared education, political affiliation, and organisational relationships, and found a strong link between government members and relevant firms.

In Portugal, the government is organised around the Prime Minister within a semi-presidential framework. Following legislative elections, the President of the Republic appoints the Prime Minister (after the constitutionally required consultations and taking the election results into account). The Prime Minister leads the Government and is responsible for its overall political orientation and coordination. Ministers and Secretaries of State are then appointed by the President upon the Prime Minister’s proposal: Ministers head a ministry (e.g., Justice, Health), while Secretaries of State support Ministers by overseeing more specialised policy areas within each ministry [8].

Beyond the constitutionally defined roles, executive governance also relies on an extensive layer of personnel within the private offices (i.e., ministerial cabinets). These consist of structures that provide direct support to the formal government representatives. Their staff varies substantially in terms of responsibilities, ranging from direct involvement in decision-making – coined as “Advisory Staff” in [5] – to organisational and operational support (i.e., “Support Staff”). The literature suggests that appointment processes are shaped by partisan considerations and trust relations within the governing party. [9] shows that appointments to ministerial cabinets in Portugal rely not only on personal trust but also on partisan loyalty, thereby reinforcing the incumbent party’s control over policy-making. Historical evidence is consistent with this pattern: Reference [10] finds that most Portuguese ministers (1851–1999) had partisan leadership experience. At the same time, they argue that although Parliament remains a major career route into ministerial office, a parliamentary background appears relatively less salient for more technical portfolios.

Mobility can also be attributed to relational dynamics. In a different institutional setting, past authors (See Reference [11]) have shown that multiplex social ties – built through co-membership in Roman Curia bodies and co-consecration relations – help characterize elite selection processes in the Catholic Church, and that some actors occupy network positions that are structurally advantageous in the context of papal elections. Inspired by this type of evidence, and knowing that members of the Portuguese political elite are tightly knit through partisan affiliation, participation in the same organizations, and educational background [7], we explore whether social connections may also be associated with ministerial appointment.

Thus, we seek to extend our work in [5] on studying the mobility of Portuguese government members by adopting a social network analysis approach. [5] examined career mobility through two complementary network types: an occupational network (where nodes are positions such as Minister or Technical Consultant, and edges reflect shared careers across those positions) and a policy-domain network (where nodes are policy-domains such as Justice or Finance, and edges capture cross-domain personnel exchanges). The primary focus was on identifying structural mobility patterns and positional centrality across the governmental hierarchy, using data on government and private office staff from 2011 to 2025. The present paper differs in three fundamental respects. First, we shift the unit of analysis from roles and domains to individuals, constructing an individual-level social network from co-participation in institutional venues. Second, we substantially expand the data: in addition to government records, we incorporate data from the Portuguese Parliament and from the Registers of Interests of public officeholders, which include private-sector ties. Third, we address a distinct research question: not patterns of career mobility across positions, but rather whether an individual’s relational position within the network is associated with subsequent appointment to ministerial office. The distributional comparison between ministers and non-ministerial actors, the categorical decomposition by venue type, and the portfolio-level retrospective screening exercise are all analytical contributions absent from [5]. In sum, our work asks whether, as in the Conclave studied by [11], relational position within the Portuguese governmental network is associated with ministerial selection. Crucially, we approach this question as an exploratory, retrospective analysis: we do not claim to predict appointments, but rather to examine whether network centrality is informative about who, among those already embedded in the network, was subsequently appointed. To that end, and acknowledging that Portuguese democracy operates within a multi-party system that has nevertheless been historically dominated by two major parties [12] – the Socialist Party (PS), a centre-left party affiliated with the Progressive Alliance of Socialists and Democrats in the European Parliament, and the Social Democratic Party (PSD), a centre-right party affiliated with the European People’s Party – we focus on the last three PS-led governments under Prime Minister António Costa: the XXI, XXII, and XXIII Constitutional Governments.

Within each policy-domain, we seek to identify which actors were structurally well-positioned to be appointed as Ministers in the XXIII Government, based on the cumulative social networks formed during the XXI and XXII Governments. By constructing the network based on two consecutive administrations led by the same party, we approximate the relational environment from which appointments to the subsequent cabinet were most likely to be drawn.

It is important to note that appointment to ministerial office in Portugal does not formally require prior elected or governmental experience, although prior political trajectories are common. Our analytical framework nevertheless assumes that candidates emerge from within the observed governmental-parliamentary network and therefore does not capture out-of-network appointments. One limitation of our framework is that it is structurally unable to identify ministers recruited from outside the observed governmental–parliamentary network. This assumption informs the temporal scope of the analysis: restricting the period to consecutive governments led by the same party reduces – though does not eliminate – the likelihood that ministers are drawn from outside the cumulative network formed in preceding administrations. In contrast, transitions between different governing parties are more likely to introduce actors whose prior relational trajectories are not embedded in the previously observed network.

Concretely, this paper makes three contributions. First, we frame ministerial recruitment as a within-network appointment problem: using the cumulative social network of two consecutive governments (XXI–XXII), we retrospectively screen potential ministerial appointees in the subsequent cabinet (XXIII) at the portfolio level and characterise the conditions under which network-based inference is more or less informative. Second, we complement this screening exercise with a distributional comparison of institutional reach, showing how Ministers and non-ministerial actors differ in their public-venue connectivity. Third, we decompose this difference across institutional categories, clarifying which arenas concentrate ministerial connectivity. Together, these analyses provide an exploratory, reproducible network-based lens on political mobility in Portugal.

The remainder of the paper is organized as follows. We first describe the data sources and the construction of the bipartite and projected social networks, and then outline the portfolio-based screening procedure and the statistical validation strategy. We then present the screening results and the distributional and categorical comparisons between Ministers and non-ministerial actors, and conclude by discussing limitations and implications for future work.

## Materials and methods

### Materials

The data employed in this study includes information on the composition of the Portuguese government, as well as staff members of the private offices of Ministers and Secretaries of State between 26 November 2015 and 2 April 2024, corresponding to the XXI to XXIII Portuguese Constitutional governments. In addition, this study draws on data on the composition of the Portuguese Parliament and its organs (e.g., Commissions and Plenary Sessions) within the same time frame (i.e., equivalent to Legislatures XIII to XV). The collection and cleaning steps for this data are the same as described in [5]. In sum, the data was extracted from official public Portuguese government portals, people’s names were homogenized using string-similarity metrics (i.e., the Levenshtein distance [13] and Jaro-Winkler similarity [14]) and policy denominations were manually standardized to be comparable across governments. However, it is important to note that, although these pre-processing steps were carefully and thoroughly revised, there may be a small number of cases in which the same person is associated with two unique identifiers due to naming inconsistencies that were not captured. Our pre-processing approach was intentionally more conservative in this regard to avoid situations in which two distinct people are identified as the same due to name similarity. Table 1 provides a random sample of disambiguations applied.

**Table 1:** Random sample of ten name disambiguation applied during person identifier deduplication.

Canonical Name	All identified names
adao jose fonseca silva	adao jose fonseca silva; adao silva
ana sofia fernandes bettencourt	ana sofia bettencourt; ana sofia fernandes bettencourt
francisco miguel vital gomes vale cesar	francisco miguel vital gomes vale cesar; francisco cesar
pedro manuel mamede passos coelho	pedro manuel mamede passos coelho; pedro passos coelho
maria luz rosinha	maria luz gameiro beja ferreira rosinha; maria luz rosinha
nilza marilia mouzinho sena	nilza sena; nilza marilia mouzinho sena
joao pedro guimaraes goncalves pereira	joao pedro guimaraes goncalves pereira; joao goncalves pereira
jorge manuel nogueiro gomes	jorge manuel nogueiro gomes; jorge gomes
bruno jorge viegas vitorino	bruno jorge viegas vitorino; bruno vitorino
isaura leonor marques figueiredo silva pedro	isaura pedro; isaura leonor marques figueiredo silva pedro

To complement the information on how these public officeholders are linked, data from the Registers of Interests of the formal members of the Portuguese government (i.e., Ministers and Secretaries of State) and the Portuguese Parliament deputies was also extracted. The Registers of Interests consist of mandatory declarations for public officeholders that detail their professional activities, assets, and other functions to ensure transparency and prevent conflicts of interest. Under Lei n.° 52/2019 (Articles 13, 15, and 17), these declarations must be submitted within 60 days of taking office and made publicly available on the respective institutional portals [15]. These were sourced from the official Portuguese Parliament portal and their contents were extracted to provide structured data on the societies and other careers held by Portuguese Government and Parliament members and their spouses at least three years prior to their mandates.

Considering that the names of the societies are often represented with slight variations, the companies’ VAT number (also known as the tax or fiscal number) was utilized as a unique identifier. Given that most of the original Registers of Interests lacked a VAT identifier, the latter was collected in bulk via the Google Search API or through online manual searching of these. For each identified society, the API was called and the first three results returned by it were evaluated. VAT numbers were extracted from the search results’ text using text-mining procedures (more specifically, regex patterns). If the same VAT number appeared across all search results for a single society, that society was associated with that identifier. To verify robustness, a reversed procedure was also applied: the candidate VAT number was searched using the API, and only VAT linked to businesses matching the original company name were automatically validated. Any society without an automatic VAT match was assigned an identifier manually by a team member through online searches of the society name/VAT number, and subsequently reviewed by a second team member to reduce the risk of assignment errors. Formal validation of automated candidates identified an error rate of 18% (67 out of 374 automated candidates). While this procedure was designed to be conservative – preferring false negatives over false positives – a small number of residual matching errors cannot be excluded. The impact of such errors on centrality measures is expected to be marginal, since private-sector ties contribute a minority of edges in the projected network and the distributional comparison of ministers versus non-ministers (see the quantitative network assessment in Results and Discussion) explicitly excludes private-sector ties from the bipartite degree centrality calculation. Other careers and functions held by politicians were filtered to include only management and administrative roles within private entities.

## Methods

**Fig 1:**
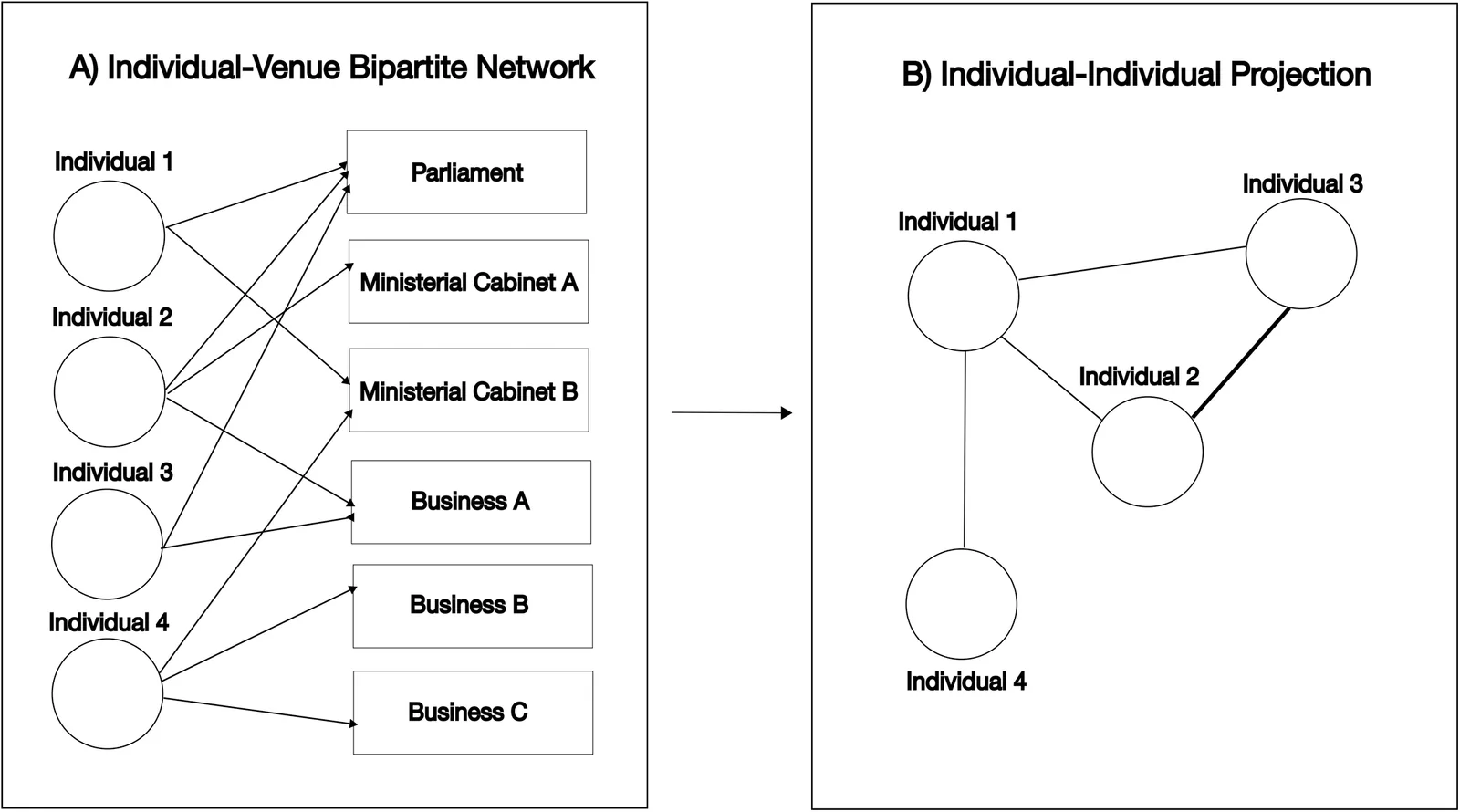
Illustration of the network construction procedure. Panel A) exemplifies the bipartite network structure, where each individual is connected to one or more venues. It is then projected into an individual-individual network (Panel B), where PEP are connected based on the number of common venues they participated in.

We first build a bipartite network linking each Political Exposed Person (PEP) to the venues they served. The two node types are: i) individuals and ii) organizational venues, including political institutions (e.g., government roles, parliamentary bodies, party structures, and Macro-area arenas) as well as private companies. An edge indicates that an individual held an official position in a given venue within a specific Government-Legislature period. We then project the bipartite network onto individuals, creating an undirected tie between two actors when they co-participate in the same venue within the same Government and Legislature (Fig 1). We estimate this cumulative network using data from the XXI and XXII Constitutional Governments, thereby capturing the relational infrastructure accumulated before the formation of the XXIII Government. Table 2 summarises the resulting network’s global properties.

**Table 2:** Global network descriptive metrics for governments XXI and XXII network.

Metric	Value
Number of Nodes	2,035
Number of Edges	159,875
Density	0.0772
Average Path Length	2.3644
Diameter	3
Edge Venue Types	Government, Macro-area, Organisations, Parliament, Parliamentary Bodies, Political Party

We acknowledge, as noted by [16], that such projections can introduce aggregation bias and that bipartite-native models offer an alternative for preserving data structure; however, for the purposes of this study, the unipartite projection remains the primary tool for identifying the latent clusters and proximity between political actors within the Portuguese executive and legislative branches.

To operationalise the idea that ministerial candidates are “influential” within the pre-appointment network, we follow [7] and flag as potential candidates the actors who rank among the top five positions in at least one node-level centrality measure. We consider rankings for betweenness (influence) [17], degree [17], weighted degree (reach) [18], closeness and weighted closeness [19], and eigenvector centrality [20], as these capture complementary dimensions of political visibility, prestige, brokerage, and accessibility within the governmental social network. This is a similar approach to that in [11], in which the Cardinals with a greater potential to be elected Pope should rank among the top 15 actors in terms of status, coalition-building capacity, and mediation power.

In this case, the combined use of degree, eigenvector, closeness, and betweenness centralities is justified by the complementary dimensions of political relevance and influence that these metrics capture within the governmental social network. Degree centrality reflects the extent of an individual’s direct connections to other deputies and government members, serving as a proxy for visibility, accessibility, and embeddedness in day-to-day political interactions. In particular, we assume that while the (unweighted) degree can serve as a measure of the reach of an individual’s direct connections, its weighted version can indicate the strength of their immediate connections (i.e., in how many different political contexts two people interact). Actors with high degree centrality are thus more likely to be known, consulted, and mobilised, which is a necessary condition for ministerial consideration. Eigenvector centrality extends this notion by weighting connections according to the importance of one’s contacts, thereby capturing positional prestige and indirect influence: individuals connected to other central and influential actors are better positioned within elite political circles, which may in turn increase their credibility and perceived suitability for a ministerial position. Betweenness centrality measures the extent to which an individual occupies brokerage positions along shortest paths between others. This can highlight an individual’s capacity to mediate between groups, policy-domains, or institutional arenas, which may also be a relevant indicator for ministerial appointment decisions. Finally, closeness centrality complements these measures by capturing how quickly an individual can reach all other actors in the network through minimal relational steps, reflecting their overall accessibility and efficiency in information diffusion.

Ministerial appointments are not easily interchangeable across policy-domains, particularly for specialised portfolios [5]. Accordingly, we also decompose the overall social network into policy-domain-specific subgraphs. Each subgraph represents the interaction context in which appointments are most likely to occur by including i) all individuals who participated in venues tagged with that policy-domain during the period of analysis and ii) their directly connected first neighbours in the projected network.

Finally, because Ministers are appointed by the Prime Minister, we restrict the centrality computation to nodes that are reachable from the Prime Minister node within each policy-domain subgraph (even if indirectly). This ensures that the screening procedure considers only actors embedded, at least minimally, in the Prime Minister’s relational sphere within the governmental network.

To assess whether our strategy of network-based identification of ministerial candidates reflects genuine structure, we compare the performance of our approach against two naive baselines: i) a random selection procedure, whereas, for each policy-domain, we simulate drawing five candidates uniformly at random from the same subgraph over 10,000 iterations, and ii) career-ladder reasoning, where ministries are filled by the most experienced former Secretaries of State in the same domain. The performance of our framework compared to these baselines is quantified through the recall – number of correctly selected ministerial appointments out of all in-network ministerial appointments.

To complement, we assess robustness of results through two sensitivity analyses. First, we experiment with different thresholds (1–15) to identify ministers among the centrality metrics and recompute recall across all baselines and our framework, holding all other parameters fixed. Secondly, we compare the base subgraph construction which expands each policy-domain subgraph to include the immediate neighbors of domain-specific nodes in the projected graph – against a strict variant, that restricts the candidate pool to nodes connected exclusively through domain-specific venue co-participation. For both settings (i.e., expanded and restricted) we report recall at *k* = 5 and the candidate-pool size per domain. Ministers present in the expanded but absent from the restricted subgraph are treated as misses under the strict condition so that both settings share a common denominator.

The study further employs a quantitative network assessment to examine whether Ministers in Government XXIII exhibit a distinct distribution of organisational connections compared to 2,478 non-ministerial individuals in that time period. By using bipartite degree centrality, the study quantifies institutional reach while intentionally excluding private-sector ties to eliminate the systemic bias caused by mandatory Registers of Interests reporting that applies only to high-ranking officials. This ensures the comparison focuses strictly on public institutional space.

To account for the significant disparity in group sizes (ministers vs. non-ministerial actors) and the highly right-skewed distribution characteristic of social network centralities [21], traditional parametric tests of mean differences are inappropriate. Instead, we employ a two-stage non-parametric validation strategy, which is the standard practice for inferential network analysis [22]. First, the Mann-Whitney U test is used to assess stochastic dominance between the groups; this rank-based test is highly robust to both severe non-normality and extreme sample size imbalances [23]. Secondly, a Permutation Test with 10,000 iterations evaluates whether ministerial connectivity is statistically unique or a product of random sampling. This allows us to assess whether Ministers occupy structurally distinct positions within the institutional network.

To complement the global metrics, we conducted a categorical structural analysis to deconstruct the minister group’s relational configuration. This approach shifts the focus from total connectivity to institutional composition, mapping influence across five categories of organisation venues: Government, Parliament, Political Party, Macro-area (i.e., policy-domain), and Parliamentary Bodies. By treating connectivity as a multi-dimensional profile, we can discern whether ministerial participation is global or concentrated within specific strategic domains.

The methodology employs a vector-based comparison, in which each individual’s centrality is normalized within each taxonomy to account for varying institutional sizes. We use cosine similarity between the mean group vectors to identify structural divergence and, once again, validate with a Permutation Test (10,000 iterations) for each category to isolate specific institutional patterns.

To complement the categorical comparison, we also construct venue-specific person-person projected networks for each of the venues and assess the centrality measures, comparing their distributions between ministers and non-ministerial actors using the same two-stage non-parametric strategy described above (permutation test with 10,000 iterations and Mann-Whitney U test). This extends beyond the question on whether ministers are connected to more venues of a given type, by studying whether they occupy structurally more central positions within the social networks formed by each institutional arena. Private-sector is excluded for the same reasons as previously described. Since restricting the projected networks to Government XXIII alone yields a single venue node per institutional category, which collapses each network into a complete graph where all actors are connected to all others and centrality scores are uniformly equal to 1, this analysis also includes all members from Governments XXI and XXII to ensure sufficient venue diversity for meaningful centrality analysis.

## Results and discussion

Of the 21 ministers appointed to the XXIII Government, including those who failed to complete their mandates and their subsequent replacements, six were absent from the policy-domain subgraphs constructed from the XXI–XXII network. These six individuals correspond to actors who had no recorded co-participation in the observed governmental or parliamentary venues during the prior two administrations, and thus represent out-of-network appointments – a category the present framework is structurally unable to recover. Characterising them falls outside our screening procedure and is itself informative: it suggests that a minority of ministerial appointments in each cabinet may reflect selection logics (e.g., technical expertise, inter-party negotiation, or direct Prime Minister preference) that operate independently of relational embeddedness in the prior network.

The remaining 15 ministers had at least one portfolio-specific subgraph entry in the XXI–XXII network. Table 3 presents the centrality rankings for these 15 ministers across their respective policy-domain subgraphs. Note that António Costa Silva appears in two separate rows because he held simultaneous responsibility over the Economy and Agriculture portfolios, which had historically been assigned to distinct ministers; each row reflects his centrality within the corresponding subgraph independently. Our approach retrospectively identifies nine of the 16 network-embedded ministerial appointments, with correct identification concentrated in stable, well-populated policy-domains.

**Table 3:** Ranking of centrality metrics for the XXIII constitutional government ministers. The last five columns indicates the overall ranking in a different centrality metric in its sub-graph: deg is the number of connections; N(SG) is the sub-graph size; Bet is the Betweenness centrality; Reach is the reach measured by the degree centrality; C is the closeness centrality of the simple graph; C(W) is the closeness centrality of the weighted graph; and EV is the eigenvector centrality.

Policy-Domain	Name	deg	N(SG)	Bet	Reach	C	C(W)	EV
Internal Administration	José Luís Pereira Carneiro	8	1411	180	165	142	282	5
Territorial Cohesion	Ana Abrunhosa	122	483	5	10	10	10	3
Agriculture, Sea, Fishing, Forestry and Rural Development	Maria Do Céu Antunes	259	1371	18	4	79	86	4
Agriculture, Sea, Fishing, Forestry and Rural Development	António Costa Silva	3	1371	576	459	245	1187	307
Economy	António Costa Silva	3	1051	399	369	247	732	370
Justice	Catarina Sarmento E Castro	3	5047	140	141	181	145	142
Infrastructures and Housing	Pedro Nuno De Oliveira Santos	462	1166	1	1	1	1	1
Infrastructures and Housing	Marina Gonçalves	395	1166	3	2	2	3	2
Infrastructures and Housing	João Saldanha De Azevedo Galamba	9	1166	113	90	87	118	93
Environment, Energy, Climate and Land Use	José Duarte Cordeiro	9	1062	269	152	131	237	168
Health	Marta Temido	3	744	5	3	3	118	4
Presidency and Administrative Modernisation	Mariana Vieira Da Silva	10	1549	63	10	5	50	5
Parliamentary Affairs	Ana Catarina Mendes	120	533	51	120	120	433	35
Labour, Solidarity and Social Security	Ana Mendes Godinho	3	1096	8	3	31	71	25
Sciences, Technology and Education	João Miguel Marques Da Costa	7	867	19	7	7	216	5
International Affairs	João Gomes Cravinho	108	884	128	108	107	130	409

A closer inspection of the ministers who were not identified as potential appointments reveals additional factors that help contextualize these outcomes. One such illustrative case is that of António Costa Silva, who was appointed minister of Economy and the Sea, thereby simultaneously holding responsibility over two distinct policy-domains – nevertheless, in the past, Constitutional Governments assigned this role to two distinct functions. To account for this institutional configuration, we extended the analysis by constructing a composite subgraph that aggregates interactions related to both portfolios. Even under this adjusted specification, António Costa Silva does not rank among the most central actors in the resulting network, suggesting that his appointment is not readily explained by relational embeddedness within the parliamentary-governmental structure.

A different logic applies to the case of João Galamba, who was appointed Minister of Infrastructure following the resignation of Pedro Nuno Santos amid a public controversy. This appointment occurred under conditions of heightened political contingency and temporal urgency, in which considerations of immediate political management, party loyalty, and continuity likely outweighed longer-term patterns of network centrality. Under such circumstances, ministerial selection is less likely to reflect structurally accumulated influence within the network and more likely to be driven by short-term strategic considerations beyond the scope of the proposed network-based identification framework.

**Fig 2:**
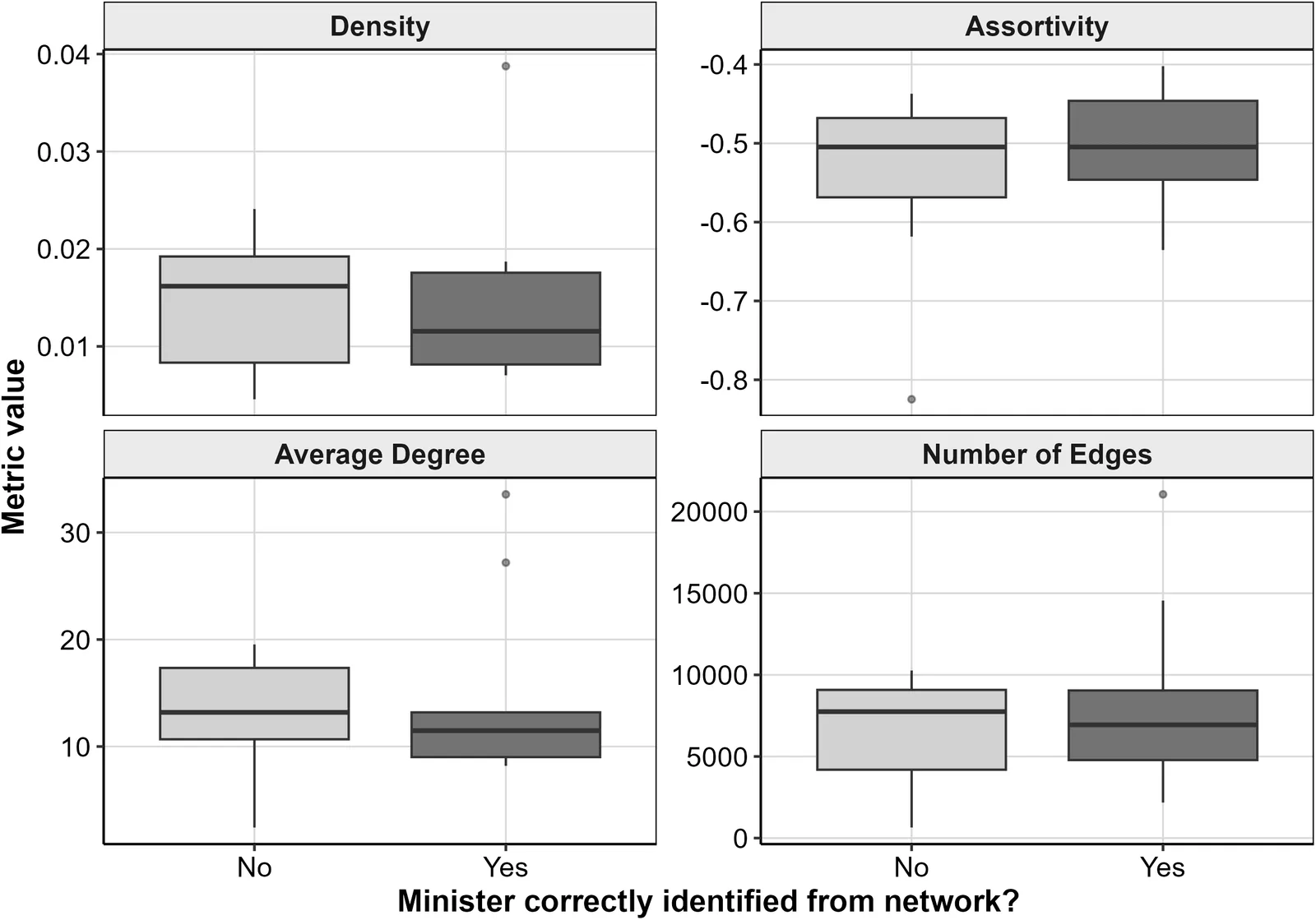
Sub-graph network metrics’ distribution by correctly identified and non-correctly identified ministerial appointments.

Examination of the structural properties of the portfolio-specific subgraphs further clarifies the conditions under which the proposed approach is more effective. Subgraphs associated with the correct identification of potential ministerial appointments exhibit relatively low average degree variability, consistently ranging from 9 to 17 connections, and are characterized by a larger number of nodes and edges. Under these conditions, the centrality measures were better able to discriminate among actors and capture meaningful differences in positional advantage (Fig 2).

**Fig 3:**
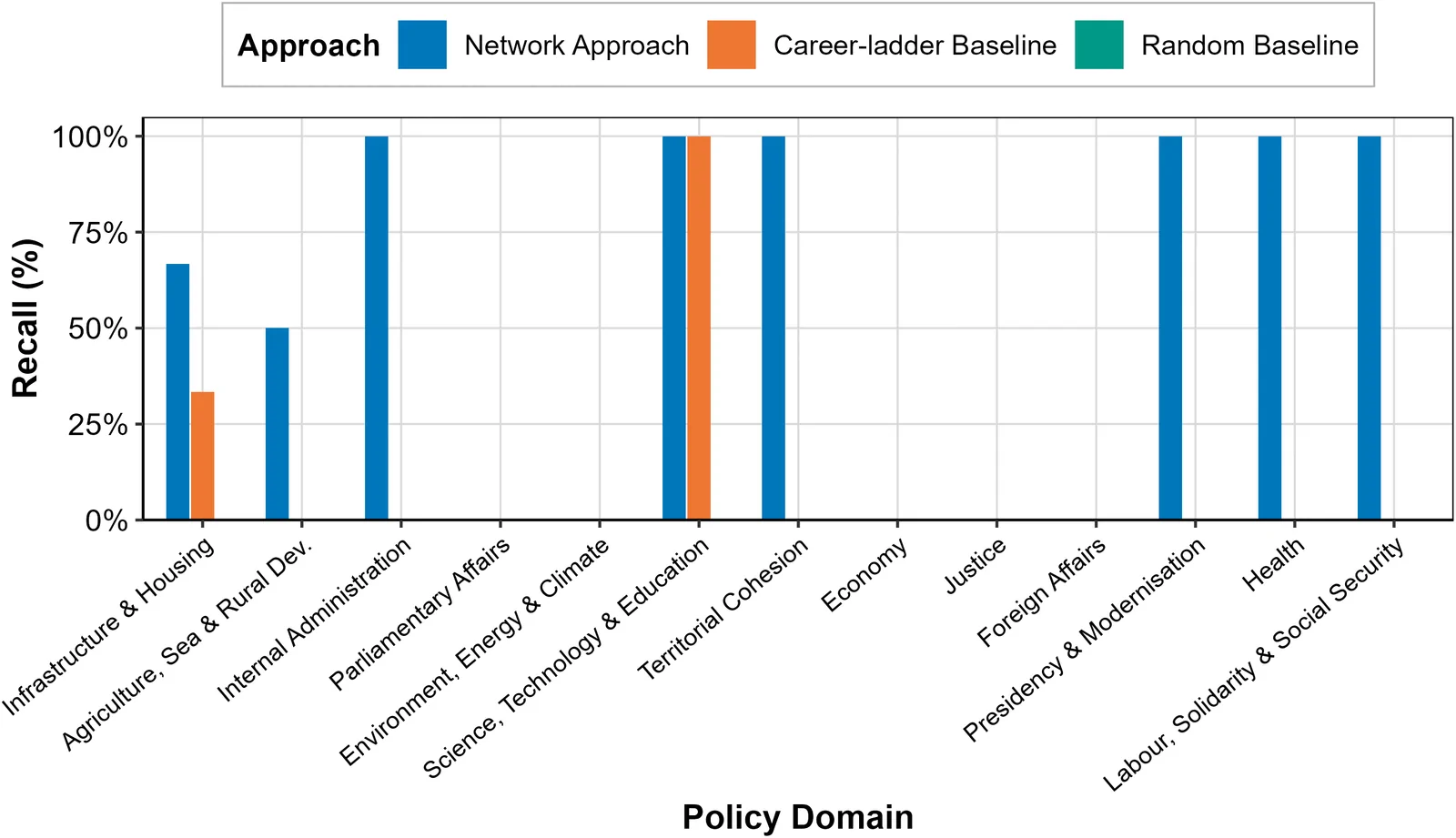
Recall performance by policy-domain against baselines.

Nevertheless, the comparison with the two baselines reinforces the validity of the network approach. From the 16 appointments embedded within the network, the baseline approach presents a recall of 0.1% to 0.8% across policy-domains (mean 0.6%), while the career-ladder heuristic yields a recall of approximately 12.5% and our approach a recall of 56.25% (Fig 3). This suggests that the model’s performance cannot be attributed to the trivial size of the candidate pool relative to the number of ministers and that network position captures information about ministerial recruitment that is not reducible to prior experience in junior executive roles within the same domain.

**Fig 4:**
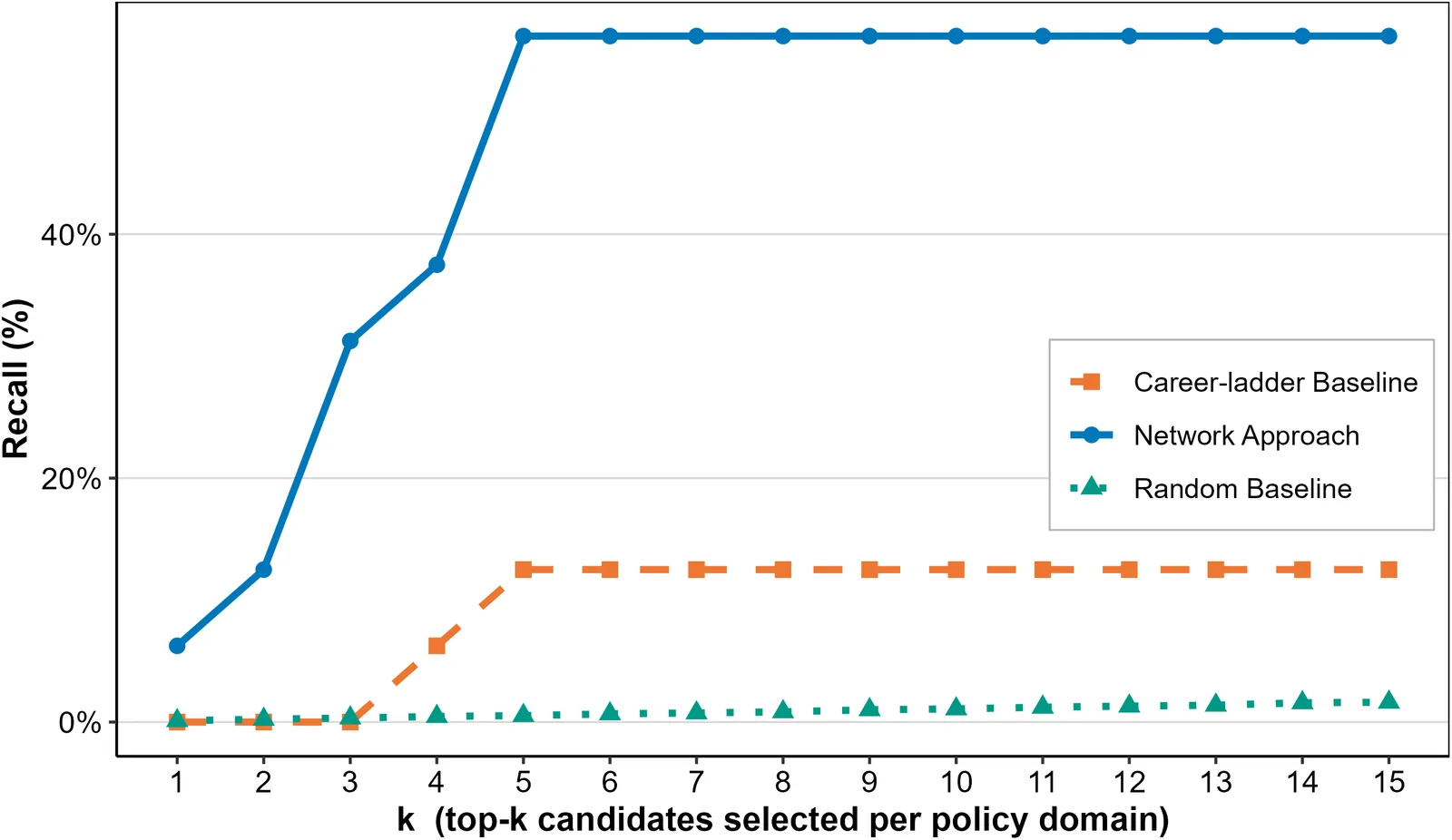
Recall across top-*k* threshold across baselines.

Sweeping the top-*k* threshold from 1 to 15 (Fig 4) shows that recall plateaus at 56.25% from *k* = 5 onward: the ministers not identified at *k* = 5 rank low across all centrality metrics and cannot be recovered by raising the threshold further. In addition, the recall curve substantially outperforms both baselines across the entire range. Restricting the candidate pool to nodes sharing a venue name that exactly matches the domain label – rather than including their first-order neighbors – reduces the mean candidate-pool size by 94.1%, yields empty subgraphs in 6 of 13 domains, and drops overall recall from 56.25% to 31.30% (Table 4), confirming that the 1-hop expansion is structurally necessary rather than a tuning choice.

**Table 4:** Candidate-pool size and recall under the expanded restricted subgraph constructions, per policy-domain. Dashes indicate domains for which the strict construction yields an empty subgraph.

policy-domain	Pool (exp.)	Pool (strict)	Recall (exp.)	Recall (strict)
Internal Administration	1,411	–	100.0%	–
Territorial Cohesion	483	40	100.0%	100.0%
Agriculture, Sea, Fishing & Forestry	1,371	131	50.0%	0.0%
Economy	1,051	–	0.0%	–
Justice	547	–	0.0%	–
Infrastructures and Housing	1,166	79	66.7%	33.3%
Environment, Energy & Climate	1,062	–	0.0%	–
Health	744	80	100.0%	100.0%
Presidency & Admin. Modernisation	1,549	187	100.0%	100.0%
Parliamentary Affairs	533	–	0.0%	–
Labour, Solidarity & Social Security	1,096	111	100.0%	0.0%
Sciences, Technology and Education	867	165	100.0%	100.0%
International Affairs	884	–	0.0%	–

**Fig 5:**
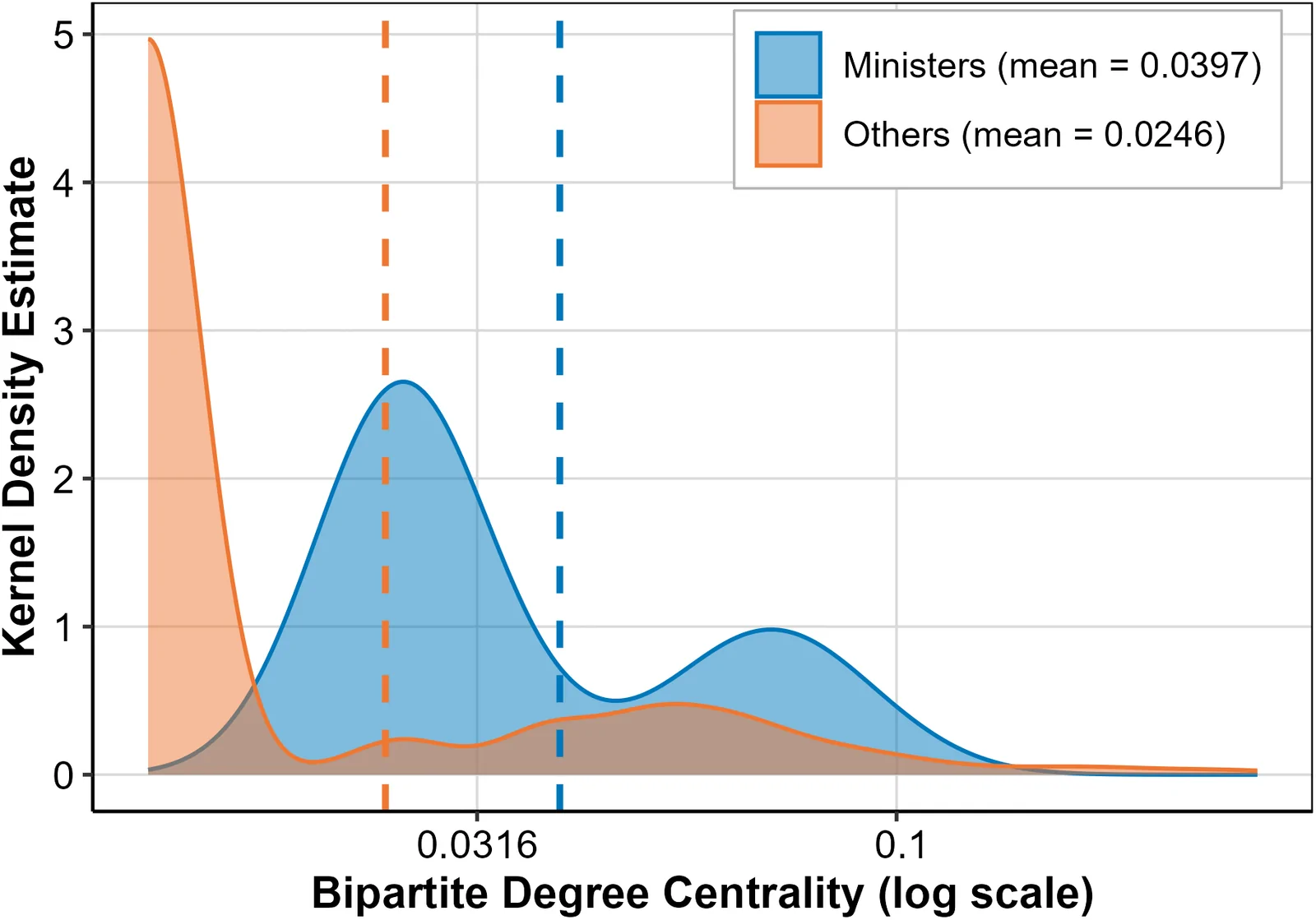
Bipartite degree centrality distributions for ministers and non-ministers (log scale). Dashed lines indicate group means.

**Fig 6:**
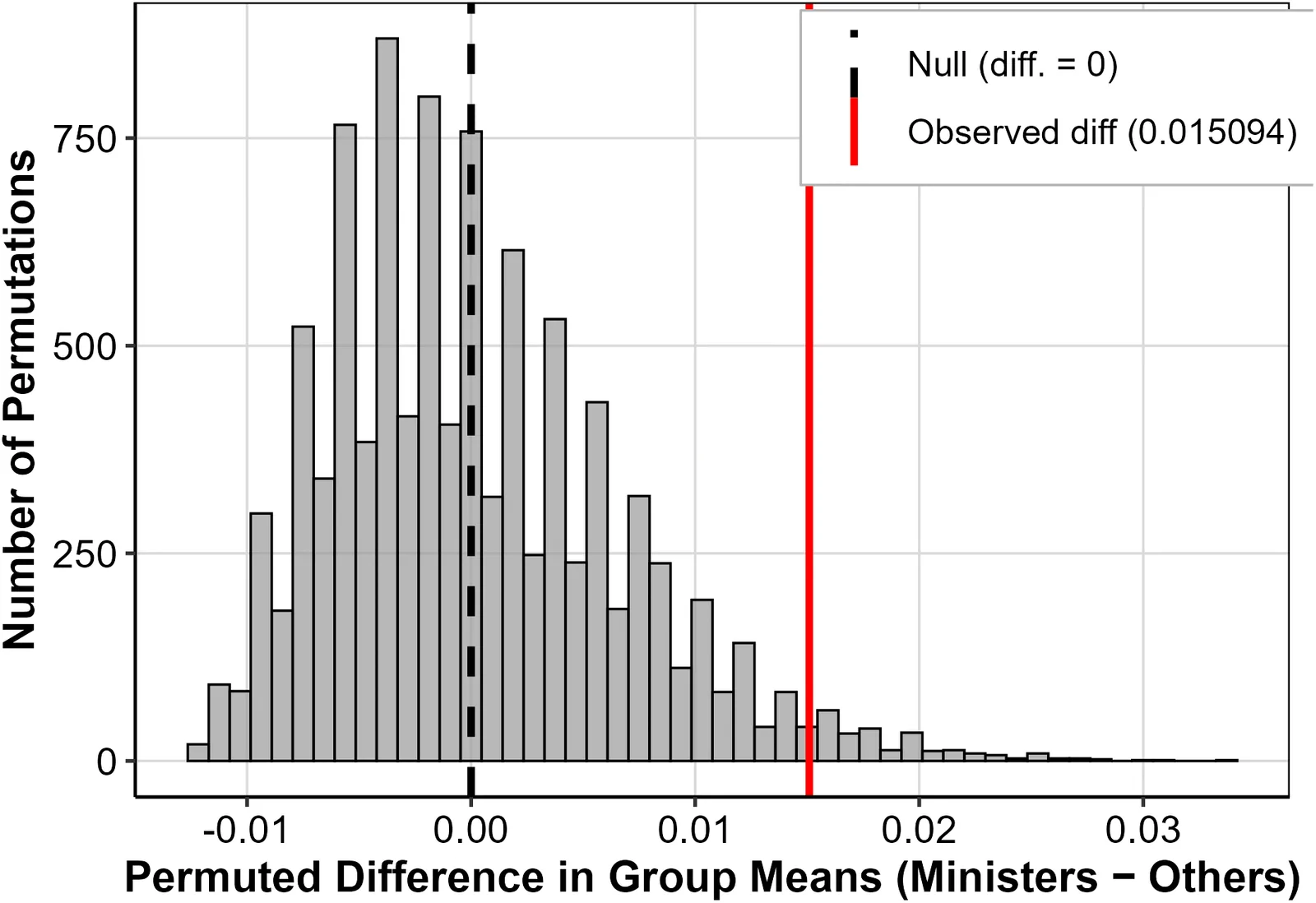
Null distribution of permuted mean centrality differences (ministers – others). The red line marks the observed difference; dashed line marks zero.

The quantitative analysis reveals a systematic difference in institutional reach between ministers and non-ministerial actors, as visualized in Figs 5 and 6. Ministers maintain a mean bipartite degree centrality 61.39% higher than their non-ministerial counterparts, a gap that is even more pronounced in the median values, where ministers exhibit double the institutional reach (0.0256 vs. 0.0128). As shown in the log-scaled KDE plot (Fig 5), the ministerial distribution is shifted toward higher centrality values, consistent with a concentration of institutional connections among this group. Due to the substantial disparity in group sizes (47 ministers versus 2,478 non-ministers), the Mann–Whitney U test was employed to ensure robust statistical inference [23]. The result (*p* < 0.001) confirms stochastic dominance of the ministerial distribution, while the permutation test (*p* = 0.021) indicates that the observed difference in means is unlikely to arise by random sampling from the full population. We note, however, that this comparison is retrospective: because the network is built from the same institutional arenas in which ministers participated, part of the observed centrality difference may reflect co-participation accumulated during (rather than before) ministerial tenure. This temporal ambiguity is an inherent limitation of the cross-sectional design and is discussed further in the limitations section.

The categorical structural analysis reveals a significant divergence in institutional engagement between Ministers and their counterparts. As statistically detailed in Table 5, the two groups yield a cosine similarity of 0.4942, confirming that Ministers and non-ministerial actors operate within fundamentally different structural logics rather than simple variations of a shared pattern. The most significant disparity is within Government venues, where Ministers maintain a mean centrality over 28 times higher than that of the “Others” group (0.5957 vs. 0.0211). This can be explained by the small group of roles that compose the Government group, namely Secretary of State, Ministers and Prime Minister.

**Table 5:** Categorical structural analysis: Mean centrality and permutation test results.

Venue Category	Ministers (Mean)	Others (Mean)	Diff	p-value
Government	0.5957	0.0211	+0.5746	0.001^***^
Parliament	0.1986	0.1071	+0.0915	0.007^**^
Political Party	0.0229	0.0124	+0.0105	0.009^**^
Macro-area	0.0388	0.0261	+0.0127	0.001^***^
Parliamentary Bodies	0.0056	0.0053	+0.0003	0.919

The statistical significance of these divergent profiles is validated by the permutation tests. The ministerial advantage is heavily concentrated within Government, Parliament, and Macro-area venues (all *p* < 0.010), where the probability of such a relational configuration arising by chance is nearly non-existent. Conversely, Parliamentary Bodies (*p* = 0.919) emerge as a point of structural convergence, suggesting a domain where ministerial status confers no significant connectivity advantage.

**Fig 7:**
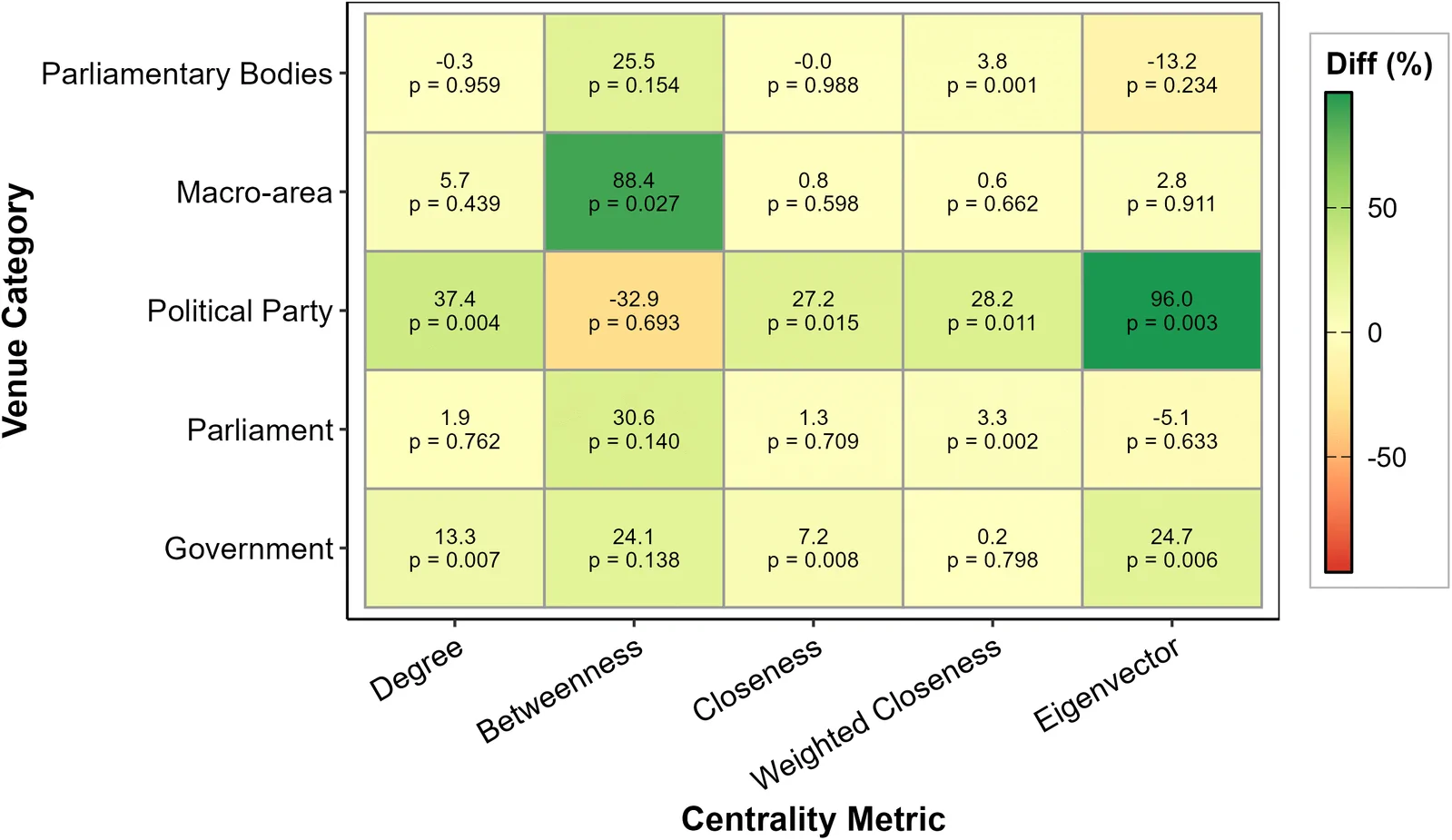
Mean centrality difference (%) between ministers and non-ministers by venue category. Cell values show the percentage difference; p-values from permutation tests are shown below.

The analysis of centrality metrics within the venue-specific networks also shows that ministerial advantage is not uniform across institutional arenas (Table 6 and Fig 7). Within the Government venue, ministers exhibit significantly higher centrality measures, consistent with ministers being better embedded and more proximate within government structures. The network on political parties yields the most consistent and substantively large minister-non-minister differences across metrics. Ministers score 37.4% higher on degree (*p* = 0.004), 27.2% higher on closeness (*p* = 0.015), 28.2% higher on weighted closeness (*p* = 0.011), and nearly double on eigenvector centrality (+96.0%; *p* = 0.003). Betweenness is the single exception, where the difference is negative and non-significant under the permutation test (*p* = 0.693). The eigenvector result indicates that ministers are not only more connected within party structures, but are connected to the most central actors within those structures, pointing to a form of elite positioning within partisan networks that substantially exceeds what proximity or breadth alone would suggest. In Parliament and policy-domain venues, the within-projection centrality differences are largely absent across most metrics; the sole exception is weighted closeness in the Parliament network (*p* = 0.002). These results suggest that the bipartite degree advantages observed for these two venue types in the categorical analysis reflect greater breadth of institutional participation rather than a structurally privileged position within the networks those venues produce. Parliamentary Bodies present a similar overall pattern: degree, betweenness, closeness, and eigenvector centrality show no meaningful minister-non-minister gap; however, weighted closeness is significantly higher among ministers (*p* < 0.001), indicating more intensive co-participation in shared parliamentary body venues even where structural position on other metrics is indistinguishable.

**Table 6:** Venue-Specific Centrality Analysis: Mean Centrality and Permutation Test Results. Deg = degree; Bet = betweenness; Clos = closeness; C(W) = weighted closeness; EV = eigenvector centrality. Significance: ^*^ *p* < 0.05, ^**^ *p* 0.01, ^***^ *p* < 0.001 (permutation test, 10,000 iterations).

Venue Category	Metric	Ministers	Others	Diff (%)	p-value
Government	Deg	0.7099	0.6266	+13.3	0.007^**^
	Bet	0.0037	0.0029	+24.1	0.138
	Clos	0.7936	0.7403	+7.2	0.008^**^
	C(W)	0.6749	0.6737	+0.2	0.798
	EV	0.0842	0.0675	+24.7	0.006^**^
Parliament	Deg	0.6620	0.6494	+1.9	0.762
	Bet	0.0011	0.0009	+30.6	0.140
	Clos	0.7644	0.7547	+1.3	0.709
	C(W)	0.7024	0.6801	+3.3	0.002^**^
	EV	0.0372	0.0392	−5.1	0.633
Political Party	Deg	0.3006	0.2187	+37.4	0.004^**^
	Bet	0.0010	0.0014	−32.9	0.693
	Clos	0.3897	0.3065	+27.2	0.015^*^
	C(W)	0.3763	0.2935	+28.2	0.011^*^
	EV	0.0498	0.0254	+96.0	0.003^**^
Macro-area	Deg	0.0586	0.0554	+5.7	0.439
	Bet	0.0014	0.0007	+88.4	0.027^*^
	Clos	0.4029	0.3999	+0.8	0.598
	C(W)	0.3955	0.3932	+0.6	0.663
	EV	0.0118	0.0115	+2.8	0.911
Parliamentary Bodies	Deg	0.6484	0.6503	−0.3	0.959
	Bet	0.0012	0.0010	+25.5	0.154
	Clos	0.7551	0.7554	−0.0	0.988
	C(W)	0.6854	0.6603	+3.8	<0.001^***^
	EV	0.0336	0.0386	−13.2	0.234

## Conclusion

This study applies the framework proposed in [11] to the context of ministerial appointments in Portugal’s XXIII Constitutional Government. Approached as an exploratory, retrospective analysis, the results suggest that social network centrality is associated with executive selection, although this association varies across policy-domains and institutional conditions. The portfolio-based screening retrospectively identified nine of the 16 network-embedded ministerial appointments, indicating that structural embeddedness provides informative signals in stable, well-populated policy-domains, and significantly outperforms the random and career-ladder baselines. These results are robust to the choice of top-k threshold – recall plateaus at 56.25% from *k* = 5 onward – and the core signal depends on the 1-hop subgraph expansion: restricting to strict domain-only nodes halves overall recall (31.30%) and empties six of 13 subgraphs.

The distributional comparison further shows that ministers have a mean bipartite degree centrality 61% higher than that of their non-ministerial counterparts. This difference is particularly pronounced within Government and Macro-area venues, where ministers operate under a structurally distinct connectivity pattern. Importantly, Parliamentary Bodies emerge as a point of convergence, where ministerial status confers no centrality advantage. While ministerial status does not confer a universal institutional advantage, the observed distributional differences are statistically robust and consistent with an association between relational embeddedness and executive recruitment, alongside other well-documented factors such as party loyalty and prior governmental experience. The venue-specific projected network analysis further reveals that this structural advantage is most consistent and substantively large within the political party network, where ministers score markedly higher on degree, closeness, and eigenvector centrality – the latter nearly double (+96%) compared to non-ministerial actors – suggesting elite positioning within partisan structures that goes beyond participation. These findings resonate with established accounts of ministerial recruitment in Portugal: [10] document that partisan leadership experience has historically been the most prevalent antecedent to ministerial office, while [9] shows that appointments to ministerial cabinets are driven by partisan loyalty and trust within the governing party. Our network analysis offers a structural counterpart to these qualitative accounts, indicating that the partisan channel operates not only through formal hierarchies but also through the relational fabric that emerges from sustained co-participation in party venues. Conversely, the six out-of-network ministers – appointed without detectable prior embeddedness – are consistent with the “technocratic” recruitment pathway identified by [24], in which external expertise or reputation substitutes for prior relational ties within the governmental–parliamentary network.

A primary limitation of this study arises from the fundamental difference between the “closed system” of a papal conclave described by [11] and the “open system” of ministerial appointments. Unlike the isolated environment of a conclave, government formation is subject to drastic external shifts, such as changes in the governing party. While the network structures of the PS-led XXI, XXII, and XXIII Governments provided a basis for this analysis, such networks likely lose their informative power when transitioning to a different political administration, such as the PSD-led XXIV Government. In these cases, the change in political leadership introduces new actors and relational logic that the previous administration’s network cannot account for.

A second important limitation concerns temporal endogeneity. The cumulative network used for screening is constructed from co-participations spanning the full XXI–XXII period, which includes co-participations that post-date some ministers’ initial appointments. Consequently, part of the centrality difference between ministers and non-ministerial actors may reflect institutional prominence accumulated *during* ministerial tenure rather than relational embeddedness that preceded appointment. Fully addressing this limitation requires constructing a temporally stratified network using only co-participations that pre-date each minister’s first appointment, which we identify as a priority for future work.

The model’s effectiveness is further limited by high-intensity political crises and institutional reconfigurations. Selections made under temporal urgency, such as the appointment of João Galamba, often prioritize immediate party loyalty over long-term structural centrality. Similarly, when portfolios are merged or redesigned – as seen with António Costa Silva – historical relational patterns may become irrelevant. Finally, the study’s reliance on specific network interactions in policy-domains means that individuals whose prior political activity occurred outside the captured subgraph remain invisible to the model. Because the discriminatory power of centrality metrics depends on subgraph density, the model remains most effective in stable, well-populated domains and less reliable in fragmented or rapidly evolving political environments.

Future research should also address the limitations of the unipartite projection by employing backbone extraction methods, such as the Stochastic Degree Sequence Model [22, SDSM]. The present manuscript does not apply a statistical framework to determine which projected edges are significantly stronger than expected under a random null model. Without such filtering, centrality rankings may be driven by spurious or trivially inflated co-occurrence ties rather than substantively meaningful relationships, a risk of aggregation bias noted by [16]. Applying backbone extraction would allow future work to assess whether the centrality-based signal identified here persists once trivial co-occurrence edges are removed.
